# Death from the Use of “Hair Restorer”

**Published:** 1870-08

**Authors:** John W. H. Baker

**Affiliations:** Chairman of Committee


ARTICLE VII. — *Death from the Use of "Hair Restorer."* Report of Committee of Investigation as to the Cause of the Death of Dr. J. M. Witherwax, of Davenport, Iowa. Read before the Scott County Medical Society. By JOHN W. H. BAKER, M.D., Chairman of Committee.

*Historic Symptoms.*—Something more than three years since, Dr. Witherwax was suffering from pains of an abdominal or colicky character, and at the time attributed them to malarial disease which he thought he had contracted in the South, while serving in the army during the war. He treated himself with that view of his case, and, after protracted treatment finally was somewhat improved; still he was occasionally subject to the pains in a more moderate degree for the next eighteen months. These attacks of pain were usually free from tenderness over the abdominal region, and would subside after vomiting; these symptoms, causing him at times to attribute his indisposition to something of a dyspeptic character. A little more than a year ago he was attacked again with an increase of the abdominal symptoms and these gradually grew worse until he had anæmic pallor, with evident jaundiced hue of the skin, disagreeable fetor, and bad taste in the mouth, loss of appetite, constipation, emaciation, and muscular debility. Pain in the abdomen gradually increasing, he at length became prostrated, and protracted vomiting occurred, which for several days was so persistent that hardly a particle of nourishment was retained within the stomach. So soon as the mildest nutriment was taken into the stomach it was followed by most distressing gastralgia, and within a short time was ejected, sometimes with a quantity of bile, and at other times with merely a small quantity of gastric juice or slimy mucus. Even after the most aggravated attack, when free from pain, the abdomen was not unusually tender, and never seemed uncommonly tympanitic.

The pains were paroxysmal or spasmodic. There were no evident febrile exacerbations; the increased heat of the body being only evident under the impulse of the extremely severe pains.

*Diagnostic Symptoms.*—The gradual development of the symptoms, such as the muscular debility; the peculiar character of the pains, such as the drawing, knotting, and obstruction, indicating in its changes, gastric, iliac, and colic varieties of location; the loss of appetite, and the final almost uncontrolable emesis, would and did indicate some other source of disease than the malarial influence left in his system from southern exposure and disease of several years previous. No medical authority would be consistent in attributing to such latent cause the persistency which these symptoms presented, returning, as they did, at intervals of one year in the more severe manifestations.

We therefore turned our attention in search of some other and more definite or positive cause. Having seen several cases of Colica Pictonum, and recognizing the similarity of the symptoms in this case, we were led to the inquiry as to whether our professional brother could by any means have been influenced by Saturnine Poison.

We readily ascertained that he had been in the habit of applying one of the popular "Hair Restorers," very frequently and copiously to his hair and whiskers, they being naturally almost snow white. With this application he had for a long time had his barber saturate his hair twice each week, and beside this was accustomed to keep a bottle of the same in his office, from which he would occasionally dress his whiskers, during the intervals of its regular application.

Under the directions of the editors of a London Chemical Journal, Mr. Henry Matthews, F. R. C. S., has recently made an analysis of some of the most widely advertised Hair Restorers, and finds that Sulphur, *Acetate of Lead*, and Glycerine, are the ingredients in every one of these much-lauded preparations. The editors of the *Medical and Surgical Reporter*, (Phil.,) in summing up a notice of these examinations, say: "Now it is notorious that the Acetate of Lead is an accumulative poison which in many instances has caused incurable paralysis."

"'Mrs. S. A. Allen's World's Hair Restorer,' by Mr. Matthews' analysis, contains (in an eight and one-half ounce bottle), Sulphur 75.6 grains, and an amount of Lead corresponding to 87 grains of the Acetate of Lead." "'Hall's Vegetable Sicilian Hair Restorer,' a bottle containing six fluid ounces, furnished 70.2 grains of Sulphur, mixed with Sulphate of Calcium, (milk of sulphur having evidently been used in the case,) also Lead, corresponding to 50.8 grains of the Acetate of Lead."

The several other Hair Restorers examined gave a very similar proportion of these ingredients, whether advertised as *Vegetable* or not.

Following these evidences we examined the symptoms indicative of lead poisoning, and find colic among the earliest symptoms; not acute at first, nor of long duration, but frequently returning, and at length becoming intolerably severe. The mouth is dry, generally absence of fever. Sickness of the stomach is present and sometimes vomiting, which will last for several days; abdomen drawn in toward the navel, and the sinking is more observed as the pain becomes more intense. Costiveness is very common and the alvine excretions are discharged with pain and difficulty. Prostration, emaciation, and sometimes paralysis, are the more advanced stages of its prolonged attacks.

Looking at these symptoms and comparing them with the symptoms which were present in the case of Dr. Witherwax, associated with the fact as to the application of the Saturnine Hair Lotion, and one can hardly come to any other conclusion than that the application of the wash and the symptoms can stand in no other relation to each other than as cause and effect. Under this impression Dr. W. was informed that he evidently had the symptoms of a person laboring under the influence of Lead Poison.

For the proof of the correctness of the above conclusion we have the more recent symptoms, and eventual death and post-mortem appearances to record.

About the middle of May, 1869, Dr. Witherwax was attacked with colic pains and disturbed digestion, soon followed by vomiting; these painful attacks occurred most frequently during the night time. After vomiting, the symptoms subsided in a measure, sometimes to be less severe for a day or two, when they would again return. These pains were associated with a constipated condition of the bowels, rendering it necessary for him to frequently take laxative or cathartic remedies, these remedies procuring small scybalous evacuations. At the same time he was accustomed to take an opiate in some form to relieve the pain. A peculiar icteric pallor was permanently fixed in his countenance, and his physiognomy expressed more or less an anxious appearance. Mouth dry, and tongue slightly coated; the uncomfortable taste in his mouth caused him to frequently resort to washing and gargling to remove the fetor. These symptoms gradually increased until about the 12th of June, 1869, when he became completely prostrated, and was obliged to give up his business, and became confined to his couch. His symptoms were now of an aggravated character, in all respects most severe, and the only relief from pain was derived from large doses of Morphia or inhalations of Chloroform. Vomiting of bilious matter, and the ejection of all nourishment; inactive condition of the bowels; extreme dryness of the mouth and fauces. There was very little tenderness over the abdomen, at least not such as indicated inflammation of an acute character. The pulse was only excited to unusual frequency while suffering from severe pain. The extreme prostration, with all the symptoms unchecked by medication, finally terminated his earthly existence, on the 24th day of June.

An autopsy was held about thirty hours after death, in the presence of quite a number of the profession.

In general appearance, the body was emaciated and pale, with ecchymosed spots over a portion of the abdominal surface. On exposing the abdominal contents found the peritoneum congested throughout its extent, both the omental and parietal portions. The intestines were not unusually distended with gas. Stomach, duodenum, and intestines generally, presented a dark color, much darker than usually found in inflammatory affections. Adhesive bands and general adhesive surfaces had formed between the intestines and the parietal portion of the peritoneum, on the right side; these bands and adherent surfaces were apparently of long standing, and probably date back to a severe attack of entero-peritonitis, which he had in New Orleans, during his service in the army. There was no evidence of recently deposited lymph, about the abdominal cavity, either organized or in its more fluid form. On opening the stomach, found it to contain a blackish green slime of the same character of the bilious matter vomited during the last few days of life. The mucous coat of the stomach showed some evidence of disorganization and gangrene. There was an unusual thinning of the coats of the stomach. The duodenum was congested, and exhibited more nearly evidence of recent inflammation than any other portion of the intestinal canal which was examined. Still no evidence of ulceration was found. The ilium, jejunum, cæcum and colon being examined at different points showed a considerable degree of congestion. An inflammatory condition was apparent in the glands of Bruner, which although enlarged and softened in some degree, were none of them found ulcerated. The spleen, of ordinary size, presented a bluish leaden color.

The liver was not enlarged, remaining of about the ordinary size. It presented much the appearance, externally, of having been parboiled. On opening into the substance it presented a light-ocherish color, resembling the color of Scotch or yellow snuff. The parenchyma was easily broken down under the finger, indicating softening of the organ. It resembled the condition usually described as fatty degeneration, with the exception of the enlargement and rounded edges, which are usually present but which were lacking in this case.

Gross, in his Elements of Pathology, says: "In fatty degeneration or fatty liver the organ is always larger than the healthy one, and its edges are knobby or obtuse." This was not the condition presented here; neither was there, as is usual, a fatty deposit on the surface or about the edges of the organ, the whole surface being unusually destitute of fat.

Some authors draw a distinction between adipose infiltration and true fatty degeneration, and observe that the former condition occurs more frequently than the latter. They hence conclude that in by far the greater number of cases of fatty liver they are not to be regarded as a result of mal-nutrition of the hepatic cells, but as the result of causes acting from without. Frerichs says: he "hesitates to identify the destruction of hepatic cells with fatty degeneration, but is disposed to regard an exudation process as the starting point of the disease." Rokitansky, and others, refer the destruction of the hepatic cells to the action of the bile. We hold the opinion that there may be a condition of color and modified organic change in the organ resembling fatty degeneration, but arising from causes so markedly the antipodes of what we usually see in fatty liver that it should not be termed fatty liver or fatty degeneration. In fatty liver the hypothesis is, that there is degeneration of hepatic cells, and this most frequently occurring in individuals accustomed to the indulgence of high feeding and indolence. In the case supposed to resemble a fatty degeneration of the liver tissue, we have no evidence of the degeneration of cell tissue, but a generally permeated circulation of fat globules, evidently drawn from the system by absorption, and serving the purpose of a circulating fluid, rather than a material for deposit in any special organ. We look upon the fatty liver, in its ordinary acceptation, as an increase of organized material; while we look upon its resemblance, as exhibited in the case before us, as the result of an extreme disorganizing force in the system. Of the former of these conditions, we have, as illustrative, the fatty liver of the indolent and gluttonous and the intemperate. While the latter (in resemblance of color and softened substance) occurs in cholera, yellow fever, and such exhaustive diseases as induce great emaciation, lack of digestive stomachic nutriment; hence necessitating a drain upon the body tissues, for the effort to support life. With these remarks our conclusions would naturally be that this condition found on post-mortem examination of the liver was not the special cause of death.

We will here introduce the microscopic and chemical examinations in the case as reported by the committee of investigation, which were recorded by Drs. Hazen and Cantwell.

To Dr. J. W. H. BAKER, Chairman of Committee to investigate the cause of the death of Dr. Witherwax:

*Sir:*—In conformity with the duty given me on the Committee, I have respectfully to report, that I have examined a portion of the liver, spleen, and kidney of Dr. Witherwax, both microscopically and chemically. The microscopical examination was made in conjunction with Dr. Farquaharson, (also, a member of the Committee). We found the liver to be extremely fatty —the cells gorged with oil globules; no colored granules or masses were seen.

The chemical analysis, a part of which was made in conjunction with Dr. Cantwell, (another member of the Committee,) was also carefully made. A portion of liver was obtained from Dr. French, who had preserved it in a test tube, with acetic acid. There was but a drachm of it, and the tests were delicate in their action. The second portion examined was much larger, and was obtained from Dr. Cantwell, and consisted of a portion of liver, spleen and kidney; these had been preserved in a quinine bottle with pepsin. The analysis in both portions was carried on in a similar way, but separately, and for sake of brevity can be stated as one analysis. The following is a record of the same:

The organic matter of solid and liquid was first boiled in a test tube, *then filtered.*

Liquid portion: A current of sulphuretted hydrogen was passed through for two hours, made by the action of sulphuric acid on sulphide of iron in a retort, and passed over by tube, $Pb\,O, HS = P\,b\,S + H\,O$. Filtered and digested with nitric acid. $Pb.\,S + N\,O5 = PbO.\,NO_5 + S$. Solution thus made, plus iodide potassium = yellow precipitate, $PbO, NO_5 + KI = Pb\,I + KO.\,NO_5$.

Solution plus chromate potassium=yellow precipitate, Pb O

$NO_5 + KO. CrO_3 = PbO. CrO_3 + KO. NO_5$. This was soluble in hydrate of potassium.

The solid portion that remained upon the first filtration was then examined. Mixture was evaporated to dryness, and the dry mass incinerated in a porcelain evaporating dish on a sand bath. This was boiled with water, filtered, and digested with nitric acid.

This solution the gas was passed through, as in the case of the liquid portion, and then put through the same tests, with like results. By this process, twice repeated, the analysis was made of the liver four different times, twice by *filtrates*, and twice by *incineration* and *digestion* of the solid matter.

Each analysis proved the presence of *lead* in the system, and the repeated processes confirm the fact.

I am, respectfully,

E. H. HAZEN, M. D., *Member of Committee.*

### CHEMICAL ANALYSIS, BY DR. CANTWELL.

DR. BAKER: *Sir*—As a member of the Committee (of which you are chairman) for the purpose of investigating the cause of the death of the late Dr. Witherwax, I submit to you the following synopsis of a chemical analysis which I was requested to make. At the time of the post-mortem examination Dr. French gave me a small portion of the liver, spleen and kidney. They were wrapped all together in a white cotton cloth. The same day I commenced my analysis; first of the liver, then of the kidney. As the analysis of each was the same, and for the sake of brevity, I will give the result of each together, so far as practicable.

### ANALYSIS.

I took about an ounce of the organic matter (the liver), and cut it into small portions, then put it into a porcelain dish, then poured over it a drachm and a half of hydrochloric acid, then added enough water to make a thin paste. The dish was then placed over a sand bath for one hour and a half. In the meantime a few grains of chlorate of potassa were dropped in at intervals of fifteen minutes. At the expiration of said time, the mass was of a white homogeneous character, the odor of chlorine had all passed off. I then added some more water, and set it on the sand bath to cool. After cooling it was passed through a filter, leaving a *filtrate* and *residue*. The filtrate was then taken and had sulphuretted hydrogen gas passed into it for twelve hours, which gave the following results:

Filtrate + HS for 12 hours=*PbS* + HCl, precipitate dark.

PbS. + heat & HO. $NO_5$=PbO. $NO_5$ + HS, precipitate soluble.

PbO. $NO_5$ + HO. $SO_3$=*PbO. $SO_3$* + HO. $NO_5$, precipitate white.

PbO. NO + KO. HO=*PbO. HO* + KO. $NO_5$, precipitate.

PbO. $NO_5$ + KI = *PbI* + HO. $NO_5$, precipitate yellow.

PbO. $NO_5$ + $KO_2$ Cr $O_3$=*$PbO_2$ Cr $O_3$* + $KONO_5$, precipitate yellow.

The above were all slight, but the two latter were best. Fearing the lead was not all out of the first filtrate, I applied the same tests, and in every case had faint indications of lead. Doing so with both liver and kidney, caused me to make four examinations in the wet analysis.

BLOW PIPE ANALYSIS.

I took the *residue*, and mixed it with carbonate of soda, then placed it on a piece of charcoal, which was put under the inner blow-pipe flame. The liver gave merely a slight yellow incrustation, not very satisfactory. Afterwards tried the residue from the kidney in the same manner, from which I not only procured the yellow incrustation, but two small granules. One of the granules was placed under the microscope by Dr. Hazen and myself; we then discovered a crystal which resembled *acetate of lead*. This makes in all six analyses that I have made, and, from the many and varied results which I have witnessed in making these examinations, I can say, with little hesitancy, that there was a great deal of *lead* in his system. I therefore think that it was one of the principal causes of his death.

With these remarks I most respectfully submit this report to you for your consideration.

ALONZO W. CANTWELL.

Grisolle, who wrote an essay upon lead colic some years since, has given a very correct account of this disease, illustrating the many and variable effects of this metal. He says, "Of the various accidents which result from the absorption of lead and its *different preparations* into the system, those which belong to the digestive apparatus are the most frequent."

The question now naturally arises as to the manner in which poisoning could take place in using these hair lotions. Authors admit three ways by which lead may be introduced into the system, viz.: by absorption through the skin; by inhalation through the pulmonary organs or lungs; and through the gastro-intestinal organs by direct exhibition, or swallowing. We conclude that through the first two methods the poisonous effects of the hair restorers are manifested. The use of these liquids affords not only the opportunity for absorption from the scalp surface, but also creates an atmosphere loaded with the vapor of lead and sulphur, which is constantly inhaled by the individuals in the habit of using them.

In conclusion we would say, that in many respects it might be better that the old assertion were true among mankind, that, "We cannot turn one hair black or white," or that we should answer negatively the question, "Can the leopard change his spots?"

